# Diversity of genotypes and pathogenicity of H9N2 avian influenza virus derived from wild bird and domestic poultry

**DOI:** 10.3389/fmicb.2024.1402235

**Published:** 2024-06-20

**Authors:** Qinhong Yang, Jia Ji, Jia Yang, Yongxian Zhang, Hongbin Yin, Hongyang Dai, Wei Wang, Suhua Li

**Affiliations:** ^1^College of Life Sciences, Southwest Forestry University, Kunming, China; ^2^Animal Disease Inspection and Supervision Institution of Yunnan Province, Kunming, China; ^3^The Management Bureau of Huize Black Necked Crane National Nature Reserve, Qujing, China

**Keywords:** H9N2 AIV, genotype S, genotype A, phylogenetic analysis, pathologic analysis, common crane, bar-headed goose

## Abstract

**Introduction:**

The H9N2 subtype is a predominant avian influenza virus (AIV) circulating in Chinese poultry, forming various genotypes (A-W) based on gene segment origins. This study aims to investigate the genotypic distribution and pathogenic characteristics of H9N2 isolates from wild birds and domestic poultry in Yunnan Province, China.

**Methods:**

Eleven H9N2 strains were isolated from fecal samples of overwintering wild birds and proximate domestic poultry in Yunnan, including four from common cranes (*Grus grus*), two from bar-headed geese (*Anser indicus*), and five from domestic poultry (*Gallus gallus*). Phylogenetic analysis was conducted to determine the genotypes, and representative strains were inoculated into Yunnan mallard ducks to assess pathogenicity.

**Results:**

Phylogenetic analysis revealed that five isolates from domestic birds and one from a bar-headed goose belong to genotype S, while the remaining five isolates from wild birds belong to genotype A. These bird-derived strains possess deletions in the stalk domain of NA protein and the N^166^D mutation of HA protein, typical of poultry strains. Genotype S H9N2 demonstrated oropharyngeal shedding, while genotype A H9N2 exhibited cloacal shedding and high viral loads in the duodenum. Both strains caused significant pathological injuries, with genotype S inducing more severe damage to the thymus and spleen, while genotype A caused duodenal muscle layer rupture.

**Discussion:**

These findings suggest that at least two genotypes of H9N2 are currently circulating in Yunnan, and Yunnan mallard ducks potentially act as intermediaries in interspecies transmission. These insights highlight the importance of analyzing the current epidemiological transmission characteristics of H9N2 among wild and domestic birds in China.

## 1 Introduction

H9N2 subtype avian influenza virus (AIV) was firstly isolated from turkey flocks in Wisconsin, USA, in 1966 (A/turkey/Wisconsin/1/1966(H9N2)) (Homme and Easterday, 1970). It spread extensively during the 1990′s, leading to continuous circulation among domestic chickens in various countries across Asia, the Middle East, and North Africa (Gu et al., 2017). The prevalence of the H9N2 AIV has been found to be extremely high, exceeding 10% in China, Bangladesh, Pakistan, and Egypt according to recent surveys conducted in live bird markets (LBMs) (Turner et al., 2017; Chaudhry et al., 2018; Liu et al., 2018; Peacock et al., 2019). Due to repeated reinfections of the same birds, particularly in longer-lived layers and breeders, as well as silent spread between farms and smallholdings, H9N2 AIV has been considered as a predominant subtype of AIVs circulating in poultry (Teng et al., 2016).

H9N2 AIV has undergone continuous evolution resulting in its classification into at least 23 genotypes (A-W) based on the eight gene segments (Gu et al., 2017). Notably, genotypes A, H, and S have been continued to circulate wildly in Chinese poultry. The genotype A, originating from BJ/94-like strains (represented by A/chicken/Beijing/1/1994), dominated the chicken populations in the 1990s (Liu et al., 2003); Early in the 21st century, genotype H emerged, characterized by the a gene constellation of hemagglutinin (*HA*), neuraminidase (*NA*), matrix (*M*), and non-structural protein (*NS*) genes from the genotype A and polymerase basic 2 (*PB2*), polymerase basic 1 (*PB1*), polymerase acidic (*PA*), and nucleoprotein (*NP*) genes from the F/98-like lineage (represented by A/chicken/Shanghai/F/1998), and became prevalent in poultry (Zhang et al., 2008). By 2007, the *PB2* and *M* genes of the genotype H were replaced by those from the G1-like lineage (presented by A/quail/Hong Kong/G1/1997), forming the genotype S, which has predominated since 2010 (Liu et al., 2016).

H9N2 has been detected in various bird species, including chickens, ducks, quails, pheasants, partridges, guinea fowl, pigeons, green peafowl, as well as wild birds (Guo et al., 2021; Wang et al., 2023). Wild aquatic birds, with their gastrointestinal viral tropism and cloacal route of viral shedding, are considered as the primary natural reservoir for AIV (Homme and Easterday, 1970; Yang et al., 2022). Billions of poultry birds are raised in China every year under the traditional small-scale and backyard-level raising model with limited biosecurity measures (Liu et al., 2020; Xue et al., 2023). As a result, domestic waterfowls, such as ducks and goose, can directly contact with wild birds through shared aquatic habitats contaminated by virus shedding (Liu et al., 2020; Xue et al., 2023). This facilitates the fecal-oral transmission of AIVs between domestic and wild birds. Domestic ducks in China, which often show few or no disease signs after viral infection and demonstrate surreptitious spread of viruses, are considered the “Trojan horses” of AIV transmission (Kim et al., 2009; Wang et al., 2023).

Actively monitoring the prevalence and evolution of influenza viruses will contribute to the development of prevention and control measures against the influenza epidemic. In this study, 11 isolates of H9N2 were identified in our annual surveillance for AIV in Dashanbao Black-Necked Crane National Nature Reserve and Huize Black-Necked Crane National Reserve, in Yunnan Province, China, from the fecal samples of wild birds and surrounding domestic poultry during 2021–2022 (Yang et al., 2022). The viral phylogenetic analysis revealed distinct genotypes between strains obtained from wild birds and domestic birds. The representative strains from the two genotypes exhibited varying levels of pathogenicity on the challenged mallard ducks. These results may contribute to our analysis of the current epidemiological transmission characteristics of H9N2 among wild and domestic birds in China.

## 2 Materials and methods

### 2.1 H9N2 isolates

In this study, 11 H9N2 AIV isolates were analyzed, including four from common crane (*Grus grus*), two from bar-headed goose (*Anser indicus*) and five from domestic poultry (*Gallus gallus*). These isolates had been previously identified during our AIV surveillance at the Dashanbao Black-Necked Crane National Nature Reserve and the Huize Black-Necked Crane National Reserve, in Yunnan Province, China (Yang et al., 2022). The identification procedures for the H9N2 AIV isolates were as follows: initially, fresh samples were propagated in chicken embryos, and the allantoic fluid was tested using the hemagglutination assay. Subsequently, viral RNA extracted from hemagglutination-positive allantoic fluid was used to identify AIV-positive samples through reverse transcription-polymerase chain reaction (RT-PCR) amplification of the *M* gene, using specific primers (AIF: 5′-CACCATGAGTCTTCTAACCGAG-3′, AIR: 5′-CTACTGTTGTATATGAGACCC-3′). Finally, the identified AIV strains were subtyped using *H9*-specific primers (H9F: 5′-CTYCACACAGARCACAATGG-3′ and H9R: 5′-GTCACACTTGTTGTTGTRTC-3′), in conjunction with *N2*-specific primers (N2F: 5′-AACACIGACTGGAGTGGYTAC-3′ and N2R: 5′-GGAATTCTGTRCTGGAACAC-3′) (Lee et al., 2001; Fereidouni et al., 2009). Detailed information regarding strain names, abbreviations, host species, and accession numbers in database were cataloged in Table 1.

**Table 1:** Detailed information of 11 H9N2 isolates identified in this study.

**Virus strains**	**Abbreviation**	**Location^a^**	**Host**	**Accession number^b^**
A/common crane/Zhaotong/3/2021(H9N2)	CC-3	Zhaotong	*Grus grus*	EPI_ISL_18137229
A/common crane/Zhaotong/6/2021(H9N2)	CC-6	Zhaotong	*Grus grus*	EPI_ISL_18137233
A/common crane/Zhaotong/7/2021(H9N2)	CC-7	Zhaotong	*Grus grus*	EPI_ISL_18137281
A/bar-headed goose/Huize/8/2021(H9N2)	BHG-8	Huize	*Anser indicus*	EPI_ISL_18137476
A/common crane/Zhaotong/11/2021(H9N2)	CC-11	Zhaotong	*Grus grus*	EPI_ISL_18137283
A/bar-headed goose/Zhaotong/21/2021(H9N2)	BHG-21	Zhaotong	*Anser indicus*	EPI_ISL_18137480
A/chicken/Huize/54/2021(H9N2)	CK-54	Huize	*Gallus gallus*	EPI_ISL_18137484
A/chicken/Huize/59/2021(H9N2)	CK-59	Huize	*Gallus gallus*	EPI_ISL_18137486
A/chicken/Zhaotong/68/2021(H9N2)	CK-68	Zhaotong	*Gallus gallus*	EPI_ISL_18137489
A/chicken/Huize/74/2021(H9N2)	CK-74	Huize	*Gallus gallus*	EPI_ISL_18137514
A/chicken/Huize/75/2021(H9N2)	CK-75	Huize	*Gallus gallus*	EPI_ISL_18137517

^a^The labels indicate the names of the regions where the isolates were obtained. “Huize” refers to Huize Black-Necked Crane National Nature Reserve, Yunnan Province, China, and “Zhaotong” refers to Zhaotong Dashanbao Black-Necked Crane National Nature Reserve, Yunnan Province, China.

^b^Accession number refers to the unique identifier for sequences in the GISAID database (http://platform.gisaid.org), where each accession number corresponds to all gene fragment sequences of the respective isolate.

### 2.2 Next generation sequencing

The complete genomes of the 11 H9N2 isolates were amplified across all eight segments utilizing a one-step RT-PCR protocol with the reported primers MBTuni-12 (5′-ACGCGTGATCAGCAAAAGCAGG-3′) and MBTuni-13 (5′-ACGCGTGATCAGTAGAAACAAGG-3′) (Zhou et al., 2009). Subsequent next-generation sequencing (NGS) of the amplified products was performed on an Illumina MiSeq platform (Illumina, CA, USA). Library preparation for sequencing was conducted using the Nextera XT Library Prep Kit (Illumina, CA, USA), in accordance with the manufacturer's instructions. Quality filtering of raw sequence data was conducted using Fastx Toolkit version 0.0.13 (http://hannonlab.cshl.edu/fastx_toolkit/index.html), discarding reads shorter than 20 base pairs and those containing ambiguous identities to produce clean reads. These quality-filtered clean reads were then assembled *de novo* using the CLC Genomics Workbench software version 6.0.4 (Qiagen Inc., MD, USA). The resulting single-gene assemblies were further refined with the CAP3 sequence assembly program (NCI, MD, USA). Assembled consensus sequences were analyzed against the National Center for Biotechnology Information (NCBI) non-redundant protein database using BLASTX with a threshold E-value of < 10^−5^ to identify significant alignments.

### 2.3 Phylogenetic analysis

The sequences of representative isolates from the reported Eurasian lineage, along with those closely related to the isolates examined in this study, were retrieved from the NCBI GenBank and Global Initiative on Sharing All Influenza Data (GISAID) EpiFlu databases, as detailed in Supplementary Table S1. These sequences were aligned using the online MAFFT service (Multiple Alignment using Fast Fourier Transform, https://mafft.cbrc.jp/alignment/software). Subsequently, maximum likelihood (ML) phylogenetic trees for each of the 11 AIV segments were constructed based on these alignments employing IQ-TREE software version 2.3.1 (UNIVIE, Vienna, Austria). The optimal substitution models were identified as GTR+F+G4 for *PB2, PB1, PA*, and *NP* segments; GTR+F+R2 for *HA*; GTR+F+I+G4 for *NA*; TVM+F+I+G4 for *M*; and TVM+F+G4 for *NS* segments. To evaluate the robustness of the phylogenetic trees, bootstrap resampling was conducted with 1,000 replicates. The finalized phylogenetic trees were then visualized and annotated utilizing FigTree version 1.4.4 (Edin., Edinburgh, UK).

### 2.4 Viral challenge with Yunnan mallard duck

Isolates CC-3 belonging to genotype A and CK-74 belonging to genotype S were designated for the experimental animal infection study, based on their high viral titers as determined by the 50% egg infectious dose (EID_50_). The Yunnan mallard duck, a local breed raised in backyard farms across the countryside of Yunnan Province, China, was designated as the challenging animal. One-week-old mallard ducks, sourced from a commercial duck farm where routine immunization against H9N2 was not practiced, were individually identified with numbered anklets and randomly assigned to three groups of eight: the genotype A inoculation group (treated with CC-3), the genotype S inoculation group (treated with CK-74), and the control group. The inoculation groups administered a 0.1 mL intranasal dose of allantoic fluid with an EID_50_ of 10^6.0^ per 0.2 mL, from either CC-3 or CK-74 strains. The control group received an equivalent volume of virus-free allantoic fluid. All groups had access to water and feed *ad libitum* during the study. Prior to viral infection, oropharyngeal (OP) and cloacal (CL) swab samples were collected from each duck for *M* gene RT-PCR amplification. Only ducks that tested negative for AIV were included in the animal challenge experiment.

Over a 14-day observation period, the ducks were monitored daily for clinical signs, with body weight measured before feeding. OP and CL swabs were collected in 1 mL of phosphate-buffered saline (PBS) at 1, 3, 4, 7, 10 and 14 days post inoculation (dpi) to assess viral shedding. On 4 and 14 dpi, four ducks from each group were euthanized and necropsied to examine for gross lesions. Portions of tissues from the thymus, trachea, liver, spleen, lung, kidney, duodenum, and heart were collected and frozen for histopathologic examination and viral load quantification.

### 2.5 Quantification of viral loads

To assess the viral shedding and replication of CC-3 and CK-74 strains in Yunnan mallard ducks, quantitative analyses were conducted on specimens obtained from OP and CL swabs, as well as tissue samples. Tissue specimens were homogenized in PBS to achieve a 10% weight/volume (W/V) ratio. The homogenates and swab suspensions were centrifuged at 3,500 rpm for 5 min, and 0.2 mL of the resulting supernatant was utilized for RNA extraction, followed by real-time RT-PCR (rRT-PCR). The rRT-PCR assay targeted the influenza virus *M* gene and was performed using the TaKaRa One Step PrimeScript™ III RT-qPCR Mix with UNG kit (TaKaRa, Kusatsu, Japan) on an ABI 7500 Fast real-time PCR system (Applied Biosystems, CA, USA). The primer-probe set consisted of forward primer M-F (5′-CGTCAACTTCAAGTACTC-3′), reverse primer M-R (5′-AGAGGATGCTCATGTTAC-3′), and a TaqMan probe (5′-FAM-CCTACTCAGATGGAGGCTTCTACC-TAMRA-3′).

For quantification, the levels of viral RNA were expressed as equivalents of EID_50_ (eqEID_50_), as employed in previous studies (Swietoń et al., 2020; Kye et al., 2021). Standards with known tenfold dilution viral titers ranging from 10^6.0^ to 10^0.0^ EID_50_ per 0.2 mL in egg allantoic fluid for each virus strain were prepared. Viral RNA extracted from these standards was quantified *M* gene by rRT-PCR in parallel with the experimental samples. The correlations between cycle threshold (Ct) values and viral titers expressed as log_10_ EID_50_ were established from these standards. The conversion of the sample Ct values using these correlations resulted in eqEID_50_.

### 2.6 Histopathological examination

Tissue specimens were fixed in a 4% paraformaldehyde solution, dehydrate using graded ethanol, vitrified by dimethylbenzene and subsequently embedded in paraffin. These paraffin-embedded tissue blocks were then sectioned into 5 μm slices. Following dewaxing and rehydration, the sections were stained with hematoxylin and eosin (HE). Microscopic lesions in the tissues were observed using an Olympus CX31 microscope (Olympus, Tokyo, Japan) and documented with a CCD imaging system (Motic, Fujian, China).

### 2.7 Statistical analysis

Two-way ANOVA was applied to examine variations in viral loads among OP, CL, and tissues samples between the two genotype viruses, employing GraphPad Prism software version 9.5.0 (GraphPad, CA, USA). A significance level *p* < 0.05 was adopted.

### 2.8 Ethics statement

The study involving animals received approval from the Academic Committee of Southwest Forestry University. The protocol was designated as SWFU-2022021, with approval granted on November 2, 2022.

## 3 Results

### 3.1 Phylogenetic characterization

The complete sequences for all eight gene segments of the 11 isolates were obtained, except for the *HA* and *M* segments of BHG-21, and the *M* segment of CC-6, which were not assembled into complete sequences by NGS. The identified sequences were submitted to the GISAID EpiFlu database (Table 1).

BLAST comparisons revealed that the gene sequences of five H9N2 isolates derived from domestic poultry, along with one strain from a bar-headed goose in Huize, displayed a remarkably high similarity to H9N2 strains identified in Chinese poultry from 2015 to 2022, with similarities ranging from 95.84% to 99.60% (Supplementary Table S2). In a similar vein, with the exception of the *PA* gene, the remaining seven gene sequences of the five H9N2 isolates sourced from wild birds were found to be homologous to strains isolated from domestic poultry in China between 1999 and 2012, with similarity spanning from 93.53% to 99.86%. Notably, the *PA* segments of these H9N2 AIVs shared close genetic relatedness with the corresponding segment of the precursor strain A/great bustard/Inner Mongolia/IM-E2/2012(H9N2), as similarities ranged between 98.84% and 99.16%. The detailed sequence homology analysis of the isolates is provided in Supplementary Table S2.

**Figure 1:**
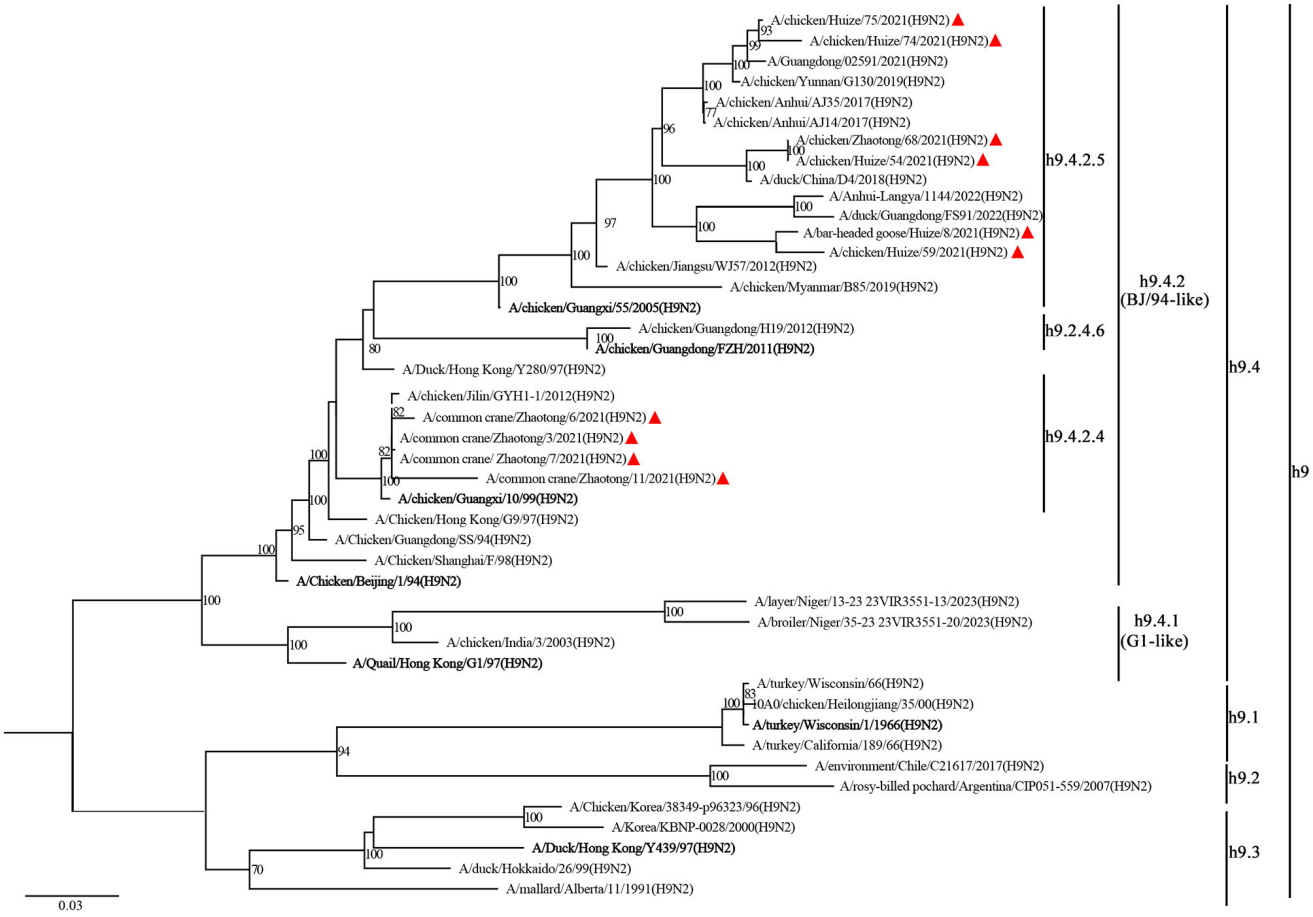
Phylogenetic analysis of the *HA* gene from H9N2 AIV. The phylogenetic tree is generated with IQ-TREE software employing the maximum likelihood (ML) method. Node support is evaluated through 1,000 bootstrap replicates, and support values exceeding 70% are indicated at the corresponding nodes. The 11 H9N2 strains sequenced in this study are denoted by red triangles, while reference sequences for each clade are emphasized in bold. Branch-specific nomenclature is detailed to the right of the tree.

The ML tree of the *HA* gene revealed that all the H9N2 isolates clustered in the same branch with the common ancestry A/Chicken/Beijing/1/94(H9N2), designated as BJ/94-like lineage or h9.4.2 lineage (Jiang et al., 2012). The h9.4.2 lineage had been further subdivided into h9.4.2.1–h9.4.2.6 (Jiang et al., 2012). The strains identified in this study were classified into two distinct sub-branches: those derived from wild birds under sub-lineage h9.4.2.4, represented by A/chicken/Guangxi/55/2005(H9N2), and those from domestic birds associated with sub-lineage h9.4.2.5, represented by A/chicken/Guangxi/10/99(H9N2) (Figure 1).

**Figure 2:**
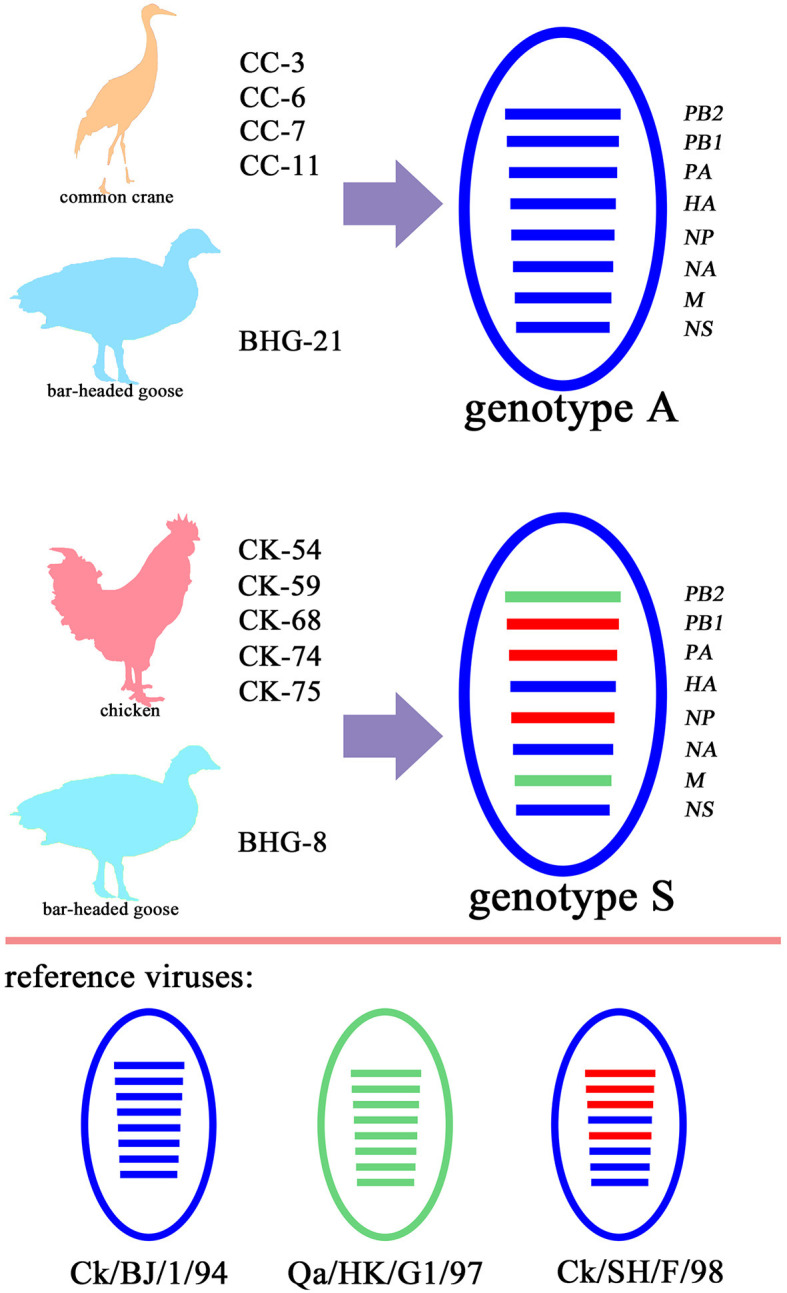
Genetic diversity of the 11 H9N2 AIV isolated in Yunnan Province, China. Four H9N2 strains isolated from common crane (CC-3, CC-6, CC-7 and CC-11) and one from bar-headed goose (BHG-21) are classified as genotype A, having acquired all eight gene segments from the BJ/94-like lineage. Conversely, five stains from chickens (CK-54, CK-59, CK-68, CK-74 and CK-75) and the one other strain from a bar-headed goose (BHG-8) are categorized as genotype S, representing a triple- reassortment derived from the BJ/94-like, G1-like, and F/98-like lineages. In the depiction, ellipses represent the AIV strains, with each of the eight horizontal segments corresponding to a distinct viral gene: polymerase basic protein 2 (*PB2*), polymerase basic protein 1 (*PB1*), polymerase acidic (*PA*), hemagglutinin (*HA*), nucleoprotein (*NP*), neuraminidase (*NA*), matrix proteins (*M*), and non-structural proteins (*NS*), respectively. Each color signifies a specific viral lineage, with BJ/94-like in blue, G1-like in green, and F/98-like in red.

The remaining seven gene segments from five wild bird-origin the H9N2 isolates were also classified within the BJ/94-like lineage (Figure 2, Supplementary Figure S1), suggesting that these isolates exhibit characteristics of genotype A, a prevalent genotype of H9N2 in Chinese domestic poultry prior to 1998. The five H9N2 isolates from domestic birds and one from a bar-headed goose (abbreviated as BHG-8) identified in this study displayed the same gene reassortment pattern. Specifically, the *HA, NA* and *NS* segments were derived from BJ/94-like lineage, *PB2* and *M* from G1-like lineage, and *PB1, PA* and *NP* segments from F/98-like lineage (Figure 2, Supplementary Figure S1). This pattern typifies the genotype S H9N2, which has been prevalent in poultry populations since 2010.

### 3.2 Key amino acid characterization

Amino acid site analysis revealed that all the isolates in this study lacked multiple consecutive basic amino acids at the HA protein cleavage site, indicating characteristics associated with low pathogenic AIV (LPAIV). Specifically, the cleavage site sequences ^333^PSRSSR↓GLF^341^ of the HA protein matched the defining feature observed in the predominant H9N2 strains circulating in China since 2013 (Heo et al., 2021). This consistent sequence pattern was observed across all genotype S isolates in this study (Table 2).

**Table 2:** Characterization of the key amino acid sites for the H9N2 AIV isolates in this study.

			**Increase the affinity to** α**-2.6-gal linked receptor (H3 numbering)**		**Enhance viral immune escape capability**		**Enhance of virus adaptability to domestic poultry**	**Enhance the activity of viral polymerase and improve the replication ability of the virus**		
		**HA**					**NA**	**PB2**	**PA**	**NP**
**Genotype**	**Virus**	**Cleavage site** ^a^	**Amino acid mutation**				**Stalk deletion** ^b^	**Amino acid mutation**		
			**Q** ^226^ **L**	**Q** ^227^ **M**	**D** ^153^ **N/G**	**N** ^166^ **D**		**I** ^292^ **V**	**K** ^356^ **R**	**I** ^353^ **V**
A	CC-3	PARSSR↓GLF	Q	Q	D	D	Yes	I	K	I
	CC-6	PARSSR↓GLF	Q	Q	D	D	Yes	I	K	I
	CC-7	PARSSR↓GLF	Q	Q	D	D	Yes	I	K	I
	CC-11	PARSSR↓GLF	Q	M	D	D	Yes	I	K	I
	BHG-21^c^	NA	NA	NA	NA	NA	Yes	I	K	I
S	BHG-8	PSRSSR↓GLF	L	M	G	D	Yes	V	R	V
	CK-54	PSRSSR↓GLF	L	M	G	N	Yes	V	R	V
	CK-59	PSRSSR↓GLF	L	M	G	D	Yes	V	K	V
	CK-68	PSRSSR↓GLF	L	M	G	D	Yes	V	R	V
	CK-74	PSRSSR↓GLF	L	M	N	D	Yes	V	R	V
	CK-75	PSRSSR↓GLF	L	M	N	D	Yes	I	R	V

^a^HA cleavage site locates in the amino acid positions 333–341 of the protein, where the host protease cleaves the HA0 precursor at the arginine residue position (indicated by the arrow), generating two subunits, HA1 and HA2.

^b^NA stalk deletion refers to the removal of amino acids at positions 63–65 in the stalk region of the NA protein.

^c^The HA segment of BHG/21 strain is deficient, therefore, “NA” is marked here.

Further analysis focused on four key amino acid sites—226, 227, 153, and 166—of the HA protein. Mutations of Q^226^L and Q^227^M (H3 numbering) were associated with altering the binding preference of HA protein to the receptors from different host species, thus facilitating interspecies transmission (Sun et al., 2021). Mutations D^153^N and N^166^D were found to enhance viral immune evasion capabilities, with the N^166^D mutation, commonly found in vaccinated domestic poultry, weakening the antibody response in avian species (Kandeil et al., 2017; Jin et al., 2019; Zhang et al., 2023). Wild bird-derived isolates belonging to genotype A typically exhibited one or two of these mutations, whereas poultry-derived isolates belonging to genotype S contained three or four mutations (Table 2). Additionally, the five wild bird-derived isolates belonging to genotype A were also found to have the deletions at positions 63–65 in the stalk region of NA protein, representing an adaptation feature for the AIVs to domestic poultry (Lv et al., 2015; Peacock et al., 2019). Isolates of genotype S originating from domestic bird demonstrated additional mutations in the polymerase proteins and NP, specifically I^292^V in the PB2, K^356^R in PA and I^353^V in NP (Table 2). Conversely, isolates derived from wild birds did not manifest these mutations. These genetic alterations may potentially augment viral replication efficiency within host cells by increasing polymerase activity, consequently contributing to increased virulence (Naffakh et al., 2008; Xu et al., 2016; Guo et al., 2022).

### 3.3 Body weight changes

None of the experimental groups reported any mortality. In the genotype S inoculation group treated with CK-74 strain, some ducks exhibited symptoms, such as coughing, mild depression, and reduced feed intake during the initial 3 dpi. In the genotype A inoculation group treated with CC-3, reduced feed intake was observed, with some ducks experiencing diarrhea starting at 4 dpi and returning to normal activity levels by 7 dpi.

**Figure 3:**
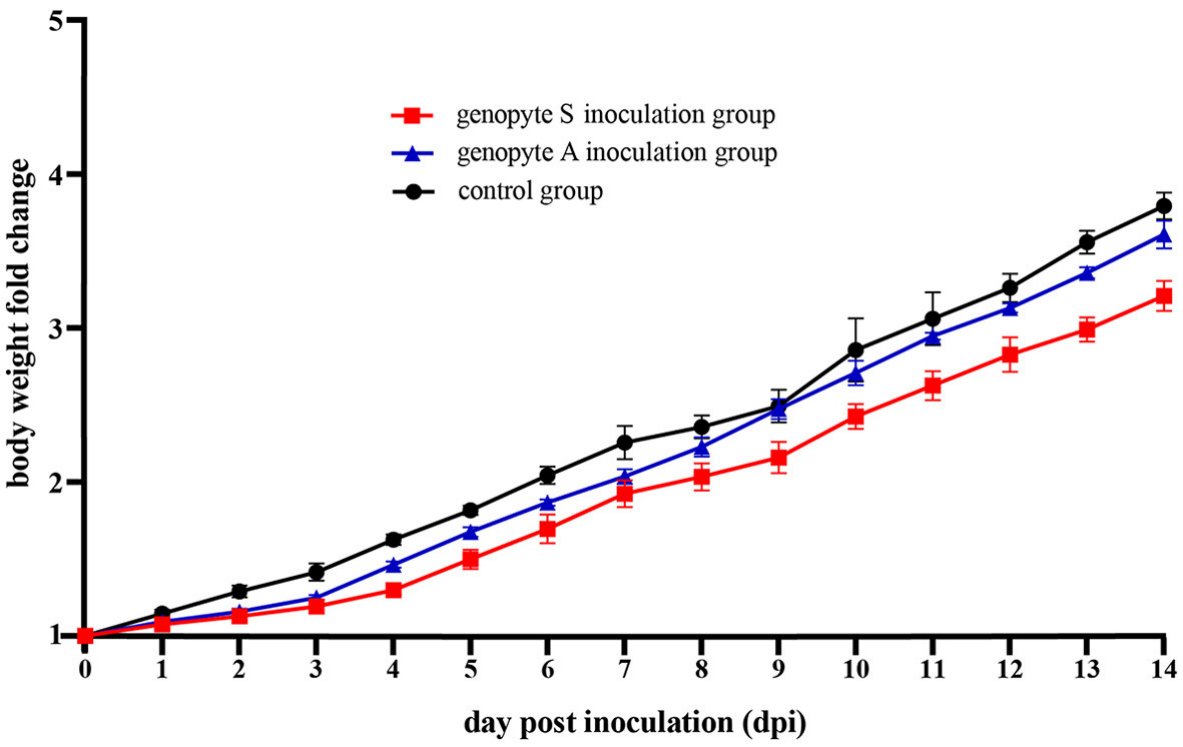
The changes in body weight of Yunnan mallard ducks challenged with H9N2 over a period of 14 dpi (days post inoculation). Body weights are graphed as the fold change, comparing them to the initial values on the day of inoculation (0 dpi). The genotype A and genotype S inoculation groups consisted of ducks intranasally inoculated with the CC-3 and CK-74 strains, respectively. The control group consisted of ducks inoculated with allantoic fluid without the virus via the same route.

Over the course of the 14-day observation period following the animal challenge experiment, ducks inoculated with H9N2 strains exhibited suppressed body weight gain in comparison to the control group (Figure 3). Moreover, ducks in genotype S inoculation group experienced less average body weight gain than those in the genotype A inoculation group. Compared to their weights on the initial inoculation date, the average body weight of ducks in the control group demonstrated a 3.79-fold increase, whereas those in the genotype A and genotype S inoculation groups exhibited increases of 3.61-fold and 3.21-fold, respectively.

Significantly, during the initial 3 days following inoculation, a pronounced suppression in body weight gain was noted (Figure 3). Specifically, on 3 dpi, the fold changes in body weight were 1.41 for the control group, 1.25 for the genotype A inoculation group, and 1.19 for the genotype S inoculation group, compared to their initial weights. Subsequently, from 4 dpi onwards, ducks in the genotype A group demonstrated body weight gains comparable to those observed in the control group. In contrast, ducks in the genotype S group began to show similar body weight gains to the control group from 9 dpi (Figure 3).

### 3.4 Viral shedding and replication characteristics

**Figure 4:**
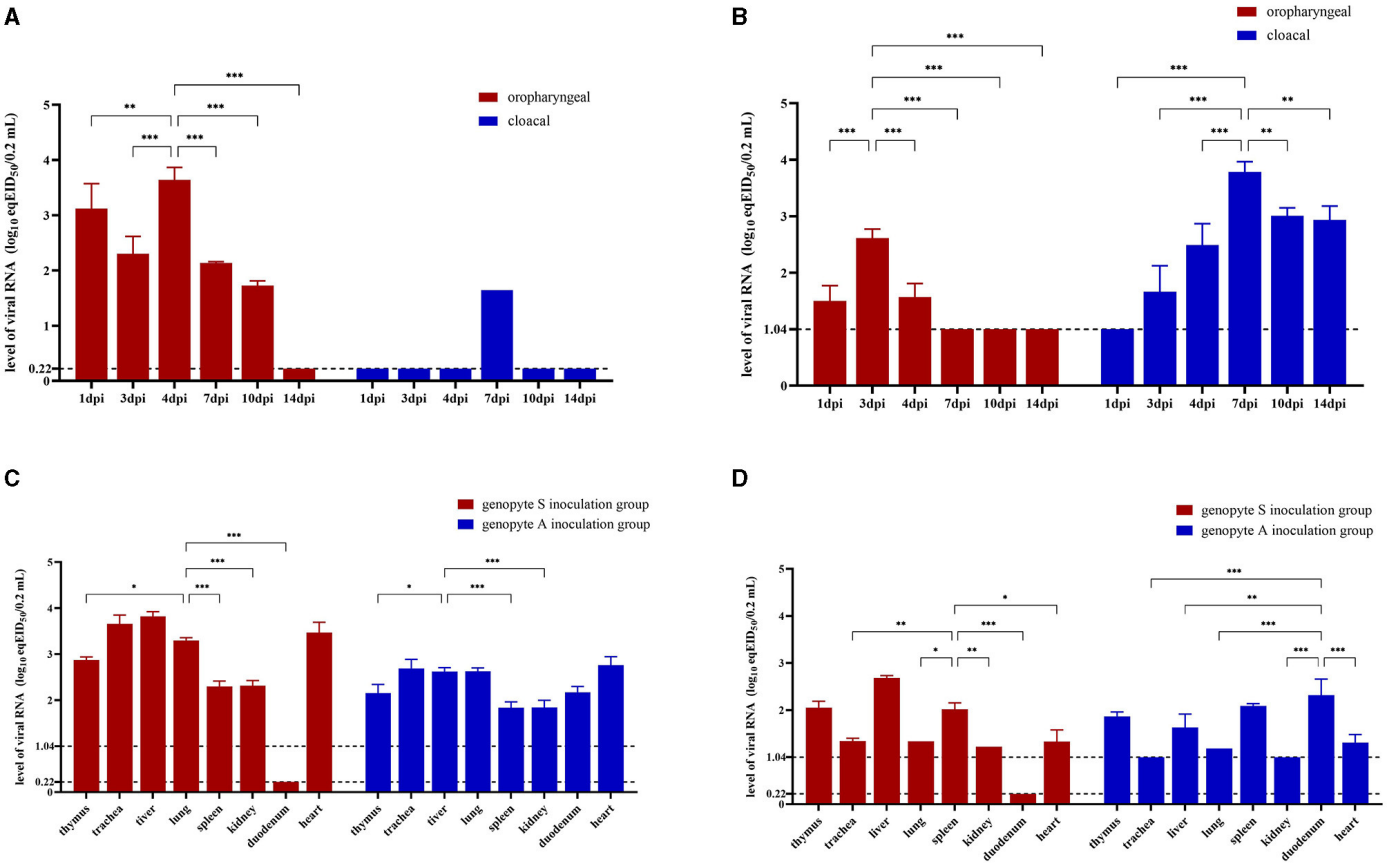
Virus loads in oropharyngeal, cloacal swabs and various organs of Yunnan mallard ducks challenged with genotype A and genotype S H9N2 AIV. Oropharyngeal and cloacal swab samples are collected from genotype S **(A)** and genotype A **(B)** H9N2 AIV inoculation groups at 1 dpi (days post inoculation), 3 dpi, 4 dpi, 7 dpi, 10 dpi, and 14 dpi. Tissue samples are obtained from four ducks in each group at 4 dpi **(C)** and 14 dpi **(D)**. The genotype A inoculation group and genotype S inoculation group are treated with CC-3 and CK-74 strains, respectively, while control group is not exposed to any virus. Virus loads are determined based on viral RNA levels expressed as equivalents of EID_50_ (eqEID_50_). The eqEID_50_ values are obtained by correlating viral titers to Ct values through rRT-PCR for the *M* gene, using viral standards with known tenfold dilution viral titers ranging from 10^6.0^ to 10^0.0^ EID_50_ per 0.2 mL in egg allantoic fluid. The standards show the limit of detection at 10^0.22^ EID_50_ for genotype S strain and 10^1.04^ EID_50_ for genotype A strain. Samples with virus loads below the limit of detection are assigned values of 0.22 log eqEID_50_ for genotype S inoculation group and 1.04 log eqEID_50_ for genotype A inoculation group, respectively. Two-way ANOVA is performed using GraphPad Prism software version 9.5.0. Significance levels are indicated by symbols: ^***^for *p* < 0.001, ^**^for *p* < 0.01, and ^*^for *p* < 0.05.

Viral shedding patterns were assessed by quantifying viral load in OP and CL swabs through rRT-PCR targeting the *M* gene. In the genotype S inoculation group treated with CK-74, the viral load in OP samples was very high on 1 dpi, slightly decreased on 3 dpi, and peaked on 4 dpi (*p* < 0.01), before declining thereafter. The CL samples in this group exhibited low viral load levels, remaining below the detectable limit except on 7 dpi. Throughout this period, the viral load in OP samples consistently exceeded that in CL samples (*p* < 0.05) (Figure 4A). Similarly, the genotype A inoculation group treated with CC-3 showed a comparable pattern in OP samples, where viral loads were with higher in the early phase (days 1, 3, and 4 dpi) compared to the later phase (days 7, 10, and 14 dpi), with a peak on 3 dpi (*p* < 0.001). In contrast, the viral load in CL samples for the genotype A group remained elevated until 7 dpi (*p* < 0.01) and was still detectable on day 14 dpi (Figure 4B).

The levels of viral RNA in tissues were used to assess the replication characteristics of the strains within the tissues. By 4 dpi, viral loads were detectable in all tissues of ducks, except for the duodenum in the genotype S inoculation group treated with CK-74 (Figure 4C). In this group, the viral loads in seven other tissues were significantly higher than those observed in the genotype A inoculation group treated with CC-3 (*p* < 0.05). Notably, the liver, heart, lungs, and trachea exhibited significantly higher viral loads compared to other tissues in both groups (*p* < 0.01). By 14 dpi, viral loads decreased across both groups. In the genotype S inoculation group, duodenum tissue continued to show undetectable viral loads (Figure 4D); however, liver, spleen, and thymus maintained higher viral loads compared to other tissues (*p* < 0.05). Conversely, in the genotype A inoculation group, while trachea and kidney tissues had undetectable viral loads at this time point, duodenum tissue retained a similar viral load as observed at 4 dpi and was significantly higher than that in trachea, liver, lung, kidney and heart (*p* < 0.01).

### 3.5 Pathological characteristics

**Figure 5:**
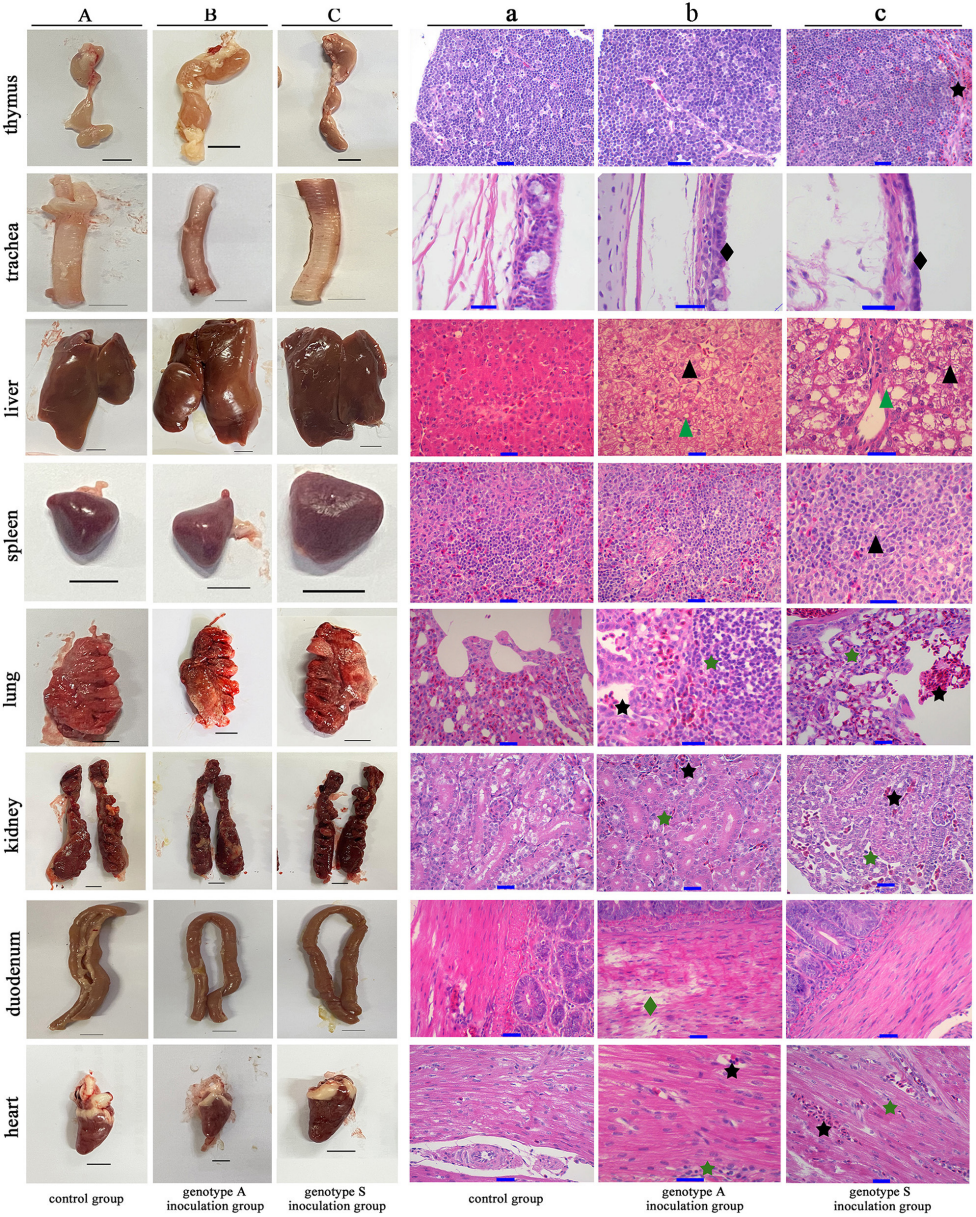
Macroscopic and microscopic pathological lesions observed in the eight tissue organs from Yunnan mallard duck challenged with genotype A and genotype S H9N2 AIV at 4 days post inoculation. **(A–C)** Illustrate the gross lesions (Scale bar = 1 cm), while **(a–c)** illustrate the corresponding microscopic lesions (Scale bar = 10 μm). Tissues presented in **(A, a)** originate from ducks in the control group, which are inoculated with virus-free chick embryo amniotic fluid. Conversely, tissues shown in **(B, b, C, c)**, are derived from ducks in the genotype S and genotype A inoculation groups, treated with CK-74 and CC-3 strains, respectively. Notable microscopic injuries are marked including hepatocellular steatosis (black triangle), hydropic degeneration (green triangle), inflammatory infiltration (green pentagram), hyperemia (black pentagram), tracheal ciliated epithelium desquamation (black diamond-shaped), and duodenal muscle layer rupture (green diamond-shaped).

Macroscopic and microscopic pathological alterations in eight tissue organs were observed at 4 dpi. Ducks from genotype S inoculation group treated with strain CK-74 exhibited extensive macroscopic pathologies, including congestion and enlargement of organs, accompanied by softening and increased fragility of the livers and lungs. Similarly, approximately 50% of the ducks in genotype A inoculation group treated with strain CC-3 demonstrated analogous macroscopic changes, such as swelling and congestion in various organs, specifically the liver, lungs, trachea, and duodenum (Figure 5).

Microscopic examination of hematoxylin-eosin-stained tissues corroborated the presence of these injuries (Figure 5). Liver tissues from both virus-inoculated groups exhibited hyperemia, hepatocellular steatosis and hydropic degeneration, characterized by cellular ballooning. Virus-inoculated ducks exhibited significant infiltration of inflammatory cells and alveolar hemorrhagic activity in the lungs, accompanied by desquamation in the tracheal pseudostratified columnar ciliated epithelium. The pathological features observed, such as inflammatory infiltration and hyperemia, were similarly evident in the renal parenchyma and cardiac muscle fibers in these virus-treated ducks. Moreover, in ducks inoculated with genotype S, atrophy and lymphocytic hydropic degeneration were noted in the spleen's white pulp, along with marked congestion in the cortical area of the thymus. Notably, intestinal tissues from ducks in genotype A group exhibited substantial damage of duodenal muscle layer rupture, a phenomenon absent in ducks from the genotype S group.

## 4 Discussion

During the 1990s, genotype A H9N2 AIV was prevalent in China. Since 2010, genotype S H9N2 has emerged as the predominant strain nationwide (Liu et al., 2016). Phylogenetic analysis of the 11 H9N2 isolates from Yunnan Province in this study reveals that both genotype A and S currently coexist in this region. Notably, genotype S is the dominant strain circulating among poultry populations, whereas genotype A is primarily found in wild birds (Figure 2). As previously reported, the genotype S isolates were characterized by a tripartite assortment, comprising the *HA, NA*, and *NS* genes from the BJ/94-like lineage, the *PB1, PA*, and *NP* genes from the F/98-like lineage, and the *PB2* and *M* genes from the G1-like lineage (Liu et al., 2016). In contrast, all eight gene segments of genotype A isolates were derived from the BJ/94-like lineage (Liu et al., 2003).

Aquatic birds, serving as natural hosts, are the primary reservoirs for transmitting AIV to domestic poultry (Spackman, 2009). Recent findings from this study have identified a genotype S strain, predominantly found in domestic poultry, within wild bar-headed geese, thereby confirming inter-species transmission of AIV between domestic and wild avian populations. Remarkably, all isolates derived from wild birds exhibited some characteristics typical of strains found in domestic birds, including a deletion of amino acids 63-65 in the stalk domain of the NA protein and the N^166^D mutation in the HA protein (Lv et al., 2015; Jin et al., 2019; Peacock et al., 2019; Zhang et al., 2023). These observations suggest a potential transmission route from domestic to wild birds within Yunnan Province.

Domestic ducks serve as an interface between wild waterfowl and terrestrial poultry, acting as potential vectors for virus transmission across species (Bi et al., 2016). In the rural areas surrounding the plateau wetlands of Yunnan Province, backyard-raised Yunnan mallard ducks frequently utilize the same water resources as wild wading birds. To investigate this interaction, we conducted viral challenge experiments using Yunnan mallard ducks exposed to genotype A and S strains of H9N2 AIV. Our findings reveal that both strains replicated in various organs and were shed via oropharyngeal or cloacal routes. These results imply that interactions between Yunnan mallard ducks and proximate wild birds may facilitate the transmission of viruses from domestic poultry to wild birds, highlighting the role of domestic ducks in the spread of avian influenza viruses.

From the animal challenge study, we also observed that the two genotypes exhibited different oropharyngeal and cloacal shedding patterns, with significant differences in viral loads in the duodenum tissue (Figure 4). The attachment of the receptor binding site (RBS) of the viral HA protein to host cell receptors is a critical step in the transmission of AIV (Imai et al., 2012). These receptors are primarily terminal sialic acids (SA) conjugated to galactose via α-2,3 or α-2,6 glycosidic bonds, forming SA α-2,3-gal and SA α-2,6-gal linked receptors, respectively. The interaction between these SA linkages and the viral HA protein exhibits specific preferences. The HA proteins of both G1-like and BJ/94-like strains, originating from avian hosts, have been reported to favor SA α-2,3-gal linked receptors (Lv et al., 2015; Peacock et al., 2017). However, the Q^226^L mutation in the viral HA protein may switch receptor binding specificity to SA α-2,6-gal linked receptors, facilitated by an increased hydrophobic environment and stronger hydrogen bond interactions (Gamblin et al., 2021). In ducks, SA α-2,3-gal linked receptors are widely present in the intestinal tract and predominate in the respiratory organs, where SA α-2,6-gal linked receptors are also distributed (Zhao and Pu, 2022). The analyzed genotype S H9N2 strain CK-74 exhibits the Q^226^L mutation in its HA protein, whereas the genotype A H9N2 strain CC-3 lacks this mutation (Table 2).

It can thus be inferred from these existing scientific observations of viral binding preferences from their HA proteins that the genotype S strain may preferentially bind to SA α-2, 6-gal linked receptors, which are expressed in the respiratory tract, whereas the genotype A strain may be inclined to bind to SA α-2, 3-gal linked receptors, primarily found in the digestive tract. This receptor specificity might explain the observed differences in virus shedding patterns, with the genotype S strain exhibiting oropharyngeal shedding and the genotype A strain displaying cloacal shedding. It is may also responsible for the continuous high viral load and the damage of muscle layer rupture in duodenal tissues of the genotype A inoculated ducks. Furthermore, this was verified that genotype A strains in aquatic birds exhibited gastrointestinal tropism and primarily spread through the oral-fecal route, whereas genotype S strains tend to show more respiratory tropism (Killingley and Nguyen-Van-Tam, 2013; Lv et al., 2015).

In this study, we observed significant lymphocyte recruitment in various internal organs, including the liver, lungs, heart, and kidneys following H9N2 AIV infection, accompanied by necrosis of hepatocytes and tracheal epithelial cells (Figure 5). A direct correlation exists between neutrophil recruitment and histopathological lesions associated with H9N2 infection in broiler chickens (Bóna et al., 2023), mice and pigeons (Short et al., 2014). During virus infection, pathogen-associated molecular patterns (PAMPs) common to various microorganisms, including AIV, are recognized by host cell pattern recognition receptors (PRRs) (Kaiser et al., 2016). This recognition triggers the host's innate immune response, producing pro-inflammatory cytokines and recruiting neutrophils, macrophages, and T lymphocytes (Kaushal, 2023). These cytokines and leukocytes create a positive feedback loop, leading to high production of proteases, reactive oxygen species (ROS), and reactive nitrogen species (RNS), which increase tissue permeability and cause cell necrosis (Short et al., 2014; Soares et al., 2023). The genotype S strain CK-74 induced more severe injury to internal organs compared to the genotype A strain CC-3. The differences in amino acid mutations between the two strains were analyzed, among which nine key mutations, including I^292^V (PB2 protein), K^356^R (PA protein), and I^353^V (NP protein) are associated with increased virulence or replication efficiency (Naffakh et al., 2008; Xu et al., 2016; Guo et al., 2022) (Table 2). These specific amino acid mutations may contribute to the pronounced pathological changes observed in the genotype S isolate. However, this hypothesis requires further experimental validation to confirm the association between these amino acid sites and pathogenicity in ducks.

In summary, we identified H9N2 AIVs from wild birds exhibiting characteristics typical of poultry strains, suggesting a possible transmission route from domestic poultry to wild birds. The Yunnan mallard duck may act as an intermediary in this transmission process. Currently, at least two genotypes of H9N2 AIV are involved in this transmission: wild bird-deriving genotype A predominantly exhibits gastrointestinal tropism and sheds virus primarily via the cloacal route, while domestic bird-deriving genotype S sheds virus mainly through the oropharyngeal (OP) route. Genotype A H9N2 AIV, which were prevalent in Chinese domestic poultry 40 years ago and are now also present in wild avian populations, remain uncertain in terms of how long they have been circulating in wild birds. Additionally, the reasons why genotype A strains did not evolve and reassort in wild birds as they did in domestic poultry remain unclear. Further research is needed to understand the dynamics of AIV transmission and evolution in different host species.

## Data availability statement

The datasets presented in this study can be found in online repositories. The names of the repository/repositories and accession number(s) can be found in the article/Supplementary material.

## Ethics statement

The animal study was approved by Academic Committee of Southwest Forestry University. The study was conducted in accordance with the local legislation and institutional requirements.

## Author contributions

QY: Software, Writing – original draft. JJ: Methodology, Writing – original draft. JY: Methodology, Software, Writing – review & editing. YZ: Resources, Writing – review & editing. HY: Resources, Writing – review & editing. HD: Resources, Writing – review & editing. WW: Software, Writing – review & editing. SL: Funding acquisition, Project administration, Supervision, Writing – review & editing.
