# Supplementary material for: Diversity of genotypes and pathogenicity of H9N2 avian influenza virus derived from wild bird and domestic poultry
Source: Front Microbiol. 2024 Jun 20;15:1402235. doi: 10.3389/fmicb.2024.1402235 (PMC11225357; doi:10.3389/fmicb.2024.1402235)
Supplement: Supplementary file 1 [file Data_Sheet_1.docx]

Supplementary Material

# Supplementary Figures and Tables

# Supplementary Table S1. Origin of the eight segment sequences of H9N2 isolates used for the phylogenetic analysis.

| **Virus strains** | **Accession number** | **Gene segment** | **Database for sequence collection^1^** |
| --- | --- | --- | --- |
| A/chicken/Guangdong/1.25_SZBJ010-O/2018(H9N2) | MW094589.1 | *PB2* | NCBI |
| A/chicken/Vietnam/HU9-506/2018(H9N2) | LC497134.1 | *PB2* | NCBI |
| A/chicken/Shandong/097/2020(H9N2) | MZ703071.1 | *PB2* | NCBI |
| A/chicken/Anhui/LH66/2017(H9N2) | MH489440.1 | *PB2* | NCBI |
| A/chicken/Zhejiang/329/2011(H9N2) | JQ356884.1 | *PB2* | NCBI |
| A/chicken/Wenzhou/3330/2013(H9N2) | KP415371.1 | *PB2* | NCBI |
| A/chicken/Qianzhou/12/2010(H9N2) | JN653565.1 | *PB2* | NCBI |
| A/chicken/Guangdong/LG1/2013(H9N2) | KC951119.1 | *PB2* | NCBI |
| A/chicken/Rizhao/437/2013(H9N2) | KF260944.1 | *PB2* | NCBI |
| A/chicken/Jiangsu/WJ57/2012(H9N2) | KP893703.1 | *PB2* | NCBI |
| A/Quail/Hong Kong/G1/97 (H9N2) | AF156435.1 | *PB2* | NCBI |
| A/chicken/Heilongjiang/35/00(H9N2) | DQ064555.1 | *PB2* | NCBI |
| A/Chicken/Hong Kong/G9/97(H9N2) | AF156430.1 | *PB2* | NCBI |
| A/chicken/Beijing/243/2010(H9N2) | KF746873.1 | *PB2* | NCBI |
| A/Duck/Hong Kong/Y280/97(H9N2) | F156433.1 | *PB2* | NCBI |
| A/Chicken/Beijing/1/94(H9N2) | AF156438.1 | *PB2* | NCBI |
| A/sparrow/Shanghai/09/2013(H9N2) | KP412429.1 | *PB2* | NCBI |
| A/chicken/Chongqing/C2093/2013(H9N2) | KM113210.1 | *PB2* | NCBI |
| A/Chicken/Shanghai/F/98(H9N2) | AY253750.1 | *PB2* | NCBI |
| A/chicken/Guangxi/55/2005(H9N2) | EU086263.1 | *PB2* | NCBI |
| A/Duck/Hong Kong/Y439/97(H9N2) | AF156434.1 | *PB2* | NCBI |
| A/American wigeon/Mexico-Sonora/769/2008(H9N2) | KY575193.1 | *PB2* | NCBI |
| A/mallard/Alberta/11/1991(H9N2) | CY005153.1 | *PB2* | NCBI |
| A/northern pintail/California/2789/2011(H9N2) | CY157525.1 | *PB2* | NCBI |
| A/Turkey/California/189/66(H9N2) | AF156443.1 | *PB2* | NCBI |
| A/turkey/Wisconsin/66(H9N2) | DQ067437.1 | *PB2* | NCBI |
| A/chicken/Xinjiang/09.19_WLMQXL026-O/2018(mixed) | MW095767.1 | *PB1* | NCBI |
| A/chicken/Yunnan/12.22_DQXBL001-C/2018(mixed) | MW095779.1 | *PB1* | NCBI |
| A/chicken/Shandong/049/2020(H9N2) | MZ703045.1 | *PB1* | NCBI |
| A/chicken/China/1102/2019(H9N2) | MN918133.1 | *PB1* | NCBI |
| A/chicken/Shantou/5500/2021(H3N8) | OQ293027.1 | *PB1* | NCBI |
| A/duck/Guangdong/FS91/2022(H9N2) | OQ826092.1 | *PB1* | NCBI |
| A/silkie chicken/Wenzhou/812/2013(H9N2) | KF260692.1 | *PB1* | NCBI |
| A/chicken/Shandong/127/2021(H9N2) | MZ703141.1 | *PB1* | NCBI |
| A/chicken/Jiangsu/WJ57/2012(H9N2) | KP893704.1 | *PB1* | NCBI |
| A/Chicken/Shanghai/F/98(H9N2) | AY253751.1 | *PB1* | NCBI |
| A/chicken/Heilongjiang/35/00(H9N2) | DQ064528.1 | *PB1* | NCBI |
| A/chicken/Guangxi/10/99(H9N2) | DQ064525.1 | *PB1* | NCBI |
| A/Quail/Hong Kong/G1/97(H9N2) | AF156421.1 | *PB1* | NCBI |
| A/Chicken/Hong Kong/G9/97(H9N2) | AF156416.1 | *PB1* | NCBI |
| A/chicken/Guangdong/6/97(H9N2) | DQ064524.1 | *PB1* | NCBI |
| A/Pigeon/Nanchang/2-0461/2000(H9N2) | CY005511.1 | *PB1* | NCBI |
| A/Duck/Hong Kong/Y280/97(H9N2) | AF156419.1 | *PB1* | NCBI |
| A/Chicken/Beijing/1/94(H9N2) | AF156423.1 | *PB1* | NCBI |
| A/American wigeon/Mexico-Sonora/769/2008(H9N2) | KY575194.1 | *PB1* | NCBI |
| A/northern pintail/California/2789/2011(H9N2) | CY157524.1 | *PB1* | NCBI |
| A/mallard/Alberta/11/1991(H9N2) | CY005152.1 | *PB1* | NCBI |
| A/Turkey/California/189/66(H9N2) | AF156429.1 | *PB1* | NCBI |
| A/turkey/Wisconsin/66(H9N2) | DQ067443.1 | *PB1* | NCBI |
| A/Duck/Hong Kong/Y439/97(H9N2) | AF156420.1 | *PB1* | NCBI |
| A/chicken/Egypt/A18504/2020(H9N2) | ON374920.1 | *PB1* | NCBI |
| A/Anhui-Langya/1144/2022(H9N2) | EPI2632294 | *PB1* | GISAID |
| A/Cambodia/21030317/2021(H9N2) | EPI2436167 | *PB1* | GISAID |
| A/Cambodia/AHC220057/2022(H9N2) | EPI2436175 | *PB1* | GISAID |
| A/common teal/Shanghai/NH111143/2021(H9N2) | EPI2058275 | *PB1* | GISAID |
| A/Bean Goose (Anser fabalis)/South Korea/KNU2021-39/2021(H9N2) | EPI2153546 | *PB1* | GISAID |
| A/layer/Niger/13-23 23VIR3551-13/2023(H9N2) | EPI2550313 | *PB1* | GISAID |
| A/broiler/Niger/35-23 23VIR3551-20/2023(H9N2) | EPI2550337 | *PB1* | GISAID |
| A/duck/Guangdong/FS91/2022(H9N2) | OQ826093.1 | *PA* | NCBI |
| A/duck/Hunan/12.27_YYGK89G3-OC/2017(H9N2) | MW108279.1 | *PA* | NCBI |
| A/chicken/Shandong/127/2021(H9N2) | MZ703142.1 | *PA* | NCBI |
| A/chicken/Vietnam/HU9-447/2018(H9N2) | LC497112.1 | *PA* | NCBI |
| A/chicken/Shandong/049/2020(H9N2) | MZ703047.1 | *PA* | NCBI |
| A/duck/Guangdong/8.30_DGCP06-O/2017(H9N2) | MW096611.1 | *PA* | NCBI |
| A/chicken/Jiangsu/WJ57/2012(H9N2) | KJ000709.1 | *PA* | NCBI |
| A/Chicken/Shanghai/F/98(H9N2) | AY253752.1 | *PA* | NCBI |
| A/great bustard/Inner Mongolia/IM-E2/2012(H9N2) | KJ907694.1 | *PA* | NCBI |
| A/Duck/Hong Kong/Y280/97(H9N2) | AF156447.1 | *PA* | NCBI |
| A/Chicken/Hong Kong/G9/97(H9N2) | AF156444.1 | *PA* | NCBI |
| A/Chicken/Beijing/1/94(H9N2) | AF156452.1 | *PA* | NCBI |
| A/Quail/Hong Kong/G1/97(H9N2) | AF156449.1 | *PA* | NCBI |
| A/chicken/Guangxi/KMIII/1999(H9N2) | HQ117885.1 | *PA* | NCBI |
| A/chicken/Heilongjiang/35/00(H9N2) | DQ064501.1 | *PA* | NCBI |
| A/sparrow/Shanghai/09/2013(H9N2) | KP412431.1 | *PA* | NCBI |
| A/chicken/Guangxi/55/2005(H9N2) | EU086258.1 | *PA* | NCBI |
| A/chicken/Fujian/G9/2009(H9N2) | JN869516.1 | *PA* | NCBI |
| A/chicken/Fujian/SIC5/2013(H9N2) | KX598612.1 | *PA* | NCBI |
| A/Duck/Hong Kong/Y439/97(H9N2) | AF156448.1 | *PA* | NCBI |
| A/Korea/KBNP-0028/2000(H9N2) | EF620899.1 | *PA* | NCBI |
| A/chicken/Egypt/A18504/2020(H9N2) | ON374921.1 | *PA* | NCBI |
| A/American wigeon/Mexico-Sonora/769/2008(H9N2) | KY575195.1 | *PA* | NCBI |
| A/environment/New Jersey/UGAI16-0887/2016(H9N2) | CY240905.1 | *PA* | NCBI |
| A/Turkey/California/189/66(H9N2) | AF156457.1 | *PA* | NCBI |
| A/turkey/Wisconsin/66(H9N2) | DQ067442.1 | *PA* | NCBI |
| A/Anhui-Langya/1144/2022(H9N2) | EPI2632295 | *PA* | GISAID |
| A/Cambodia/21030317/2021(H9N2) | EPI2436168 | *PA* | GISAID |
| A/Cambodia/AHC220057/2022(H9N2) | EPI2436176 | *PA* | GISAID |
| A/layer/Niger/13-23 23VIR3551-13/2023(H9N2) | EPI2550311 | *PA* | GISAID |
| A/broiler/Niger/35-23 23VIR3551-20/2023(H9N2) | EPI2550335 | *PA* | GISAID |
| A/Chicken/Bangladesh/55069/2022(H9N2) | EPI2397306 | *PA* | GISAID |
| A/chicken/Egypt/Menoufia/2021(H9N2) | EPI2175831 | *PA* | GISAID |
| A/Bean Goose (Anser fabalis)/South Korea/KNU2021-39/2021(H9N2) | EPI2153547 | *PA* | GISAID |
| A/common teal/Shanghai/NH111143/2021(H9N2) | EPI2058274 | *PA* | GISAID |
| A/chicken/Anhui/AJ35/2017(H9N2) | MK544456.1 | *HA* | NCBI |
| A/chicken/Anhui/AJ14/2017(H9N2) | MK544453.1 | *HA* | NCBI |
| A/duck/China/D4/2018(H9N2) | MN384776.1 | *HA* | NCBI |
| A/duck/Guangdong/FS91/2022(H9N2) | OQ826094.1 | *HA* | NCBI |
| A/chicken/Jiangsu/WJ57/2012(H9N2) | KJ000710.1 | *HA* | NCBI |
| A/chicken/Myanmar/B85/2019(H9N2) | OQ507814.1 | *HA* | NCBI |
| A/chicken/Guangxi/55/2005(H9N2) | EU086245.1 | *HA* | NCBI |
| A/chicken/Guangdong/H19/2012(H9N2) | KJ768986.1 | *HA* | NCBI |
| A/chicken/Guangdong/FZH/2011(H9N2) | JF715024.1 | *HA* | NCBI |
| A/Duck/Hong Kong/Y280/97(H9N2) | AF156376.1 | *HA* | NCBI |
| A/chicken/Jilin/GYH1-1/2012(H9N2) | KF886497.1 | *HA* | NCBI |
| A/chicken/Guangxi/10/99(H9N2) | DQ064363.1 | *HA* | NCBI |
| A/Chicken/Hong Kong/G9/97(H9N2) | AF156373.1 | *HA* | NCBI |
| A/Chicken/Guangdong/SS/94(H9N2) | AF384557.1 | *HA* | NCBI |
| A/Chicken/Shanghai/F/98(H9N2) | AY743216.1 | *HA* | NCBI |
| A/Chicken/Guangdong/SS/94(H9N2) | AF384557.1 | *HA* | NCBI |
| A/chicken/India/3/2003(H9N2) | JX273542.1 | *HA* | NCBI |
| A/Quail/Hong Kong/G1/97(H9N2) | AF156378.1 | *HA* | NCBI |
| A/turkey/Wisconsin/66(H9N2) | DQ067444.1 | *HA* | NCBI |
| A/chicken/Heilongjiang/35/00(H9N2) | DQ064366.1 | *HA* | NCBI |
| A/turkey/Wisconsin/1/1966(H9N2) | CY130054.1 | *HA* | NCBI |
| A/turkey/California/189/66(H9N2) | AF156390.1 | *HA* | NCBI |
| A/environment/Chile/C21617/2017(H9N2) | MH517593.1 | *HA* | NCBI |
| A/rosy-billed pochard/Argentina/CIP051-559/2007(H9N2) | CY111590.1 | *HA* | NCBI |
| A/Korea/KBNP-0028/2000(H9N2) | EF620900.1 | *HA* | NCBI |
| A/Duck/Hong Kong/Y439/97(H9N2) | AF156377.1 | *HA* | NCBI |
| A/mallard/Alberta/11/1991(H9N2) | CY005990.1 | *HA* | NCBI |
| A/American wigeon/Mexico-Sonora/769/2008(H9N2) | KY575196.1 | *HA* | NCBI |
| A/northern pintail/California/2789/2011(H9N2) | CY157518.1 | *HA* | NCBI |
| A/Guangdong/02591/2021(H9N2) | EPI1884602 | *HA* | GISAID |
| A/Anhui-Langya/1144/2022(H9N2) | EPI2632300 | *HA* | GISAID |
| A/layer/Niger/13-23 23VIR3551-13/2023(H9N2) | EPI2550315 | *HA* | GISAID |
| A/broiler/Niger/35-23 23VIR3551-20/2023(H9N2) | EPI2550339 | *HA* | GISAID |
| A/chicken/Shandong/127/2021(H9N2) | MZ703144.1 | *NP* | NCBI |
| A/chicken/Shandong/10.23_TAWL025-O/2018(H9N2) | MW099249.1 | *NP* | NCBI |
| A/chicken/Yunnan/11.22_DQWGH007-O/2018(mixed) | MW099041.1 | *NP* | NCBI |
| A/chicken/Guangxi/8.25_NNXH008-O/2018(mixed) | MW104249.1 | *NP* | NCBI |
| A/chicken/Shandong/049/2020(H9N2) | MZ703049.1 | *NP* | NCBI |
| A/duck/Guangdong/FS91/2022(H9N2) | OQ826095.1 | *NP* | NCBI |
| A/chicken/Jiangsu/WJ57/2012(H9N2) | KP893705.1 | *NP* | NCBI |
| A/chicken/Guangxi/55/2005(H9N2) | EU086253.1 | *NP* | NCBI |
| A/Chicken/Shanghai/F/98(H9N2) | AY253753.1 | *NP* | NCBI |
| A/Duck/Hong Kong/Y439/97(H9N2) | AF156406.1 | *NP* | NCBI |
| A/quail/Shantou/11195/2005(H9N2) | EF155193.1 | *NP* | NCBI |
| A/quail/Zhejiang/2A3/2013(H9N2) | KU042306.1 | *NP* | NCBI |
| A/Quail/Hong Kong/G1/97 (H9N2) | AF156407.1 | *NP* | NCBI |
| A/quail/Shantou/1235/2001(H9N2) | EF155136.1 | *NP* | NCBI |
| A/Duck/Hong Kong/Y280/97(H9N2) | AF156405.1 | *NP* | NCBI |
| A/chicken/Jiangsu/1/00(H9N2) | DQ064453.1 | *NP* | NCBI |
| A/Chicken/Hong Kong/G9/97(H9N2) | AF156402.1 | *NP* | NCBI |
| A/Chicken/Beijing/1/94(H9N2) | AF156409.1 | *NP* | NCBI |
| A/great bustard/Inner Mongolia/IM-E2/2012(H9N2) | KJ907696.1 | *NP* | NCBI |
| A/chicken/Guangxi/10/99(H9N2) | DQ064444.1 | *NP* | NCBI |
| A/mallard/Alberta/11/1991(H9N2) | CY005149.1 | *NP* | NCBI |
| A/northern pintail/California/2789/2011(H9N2) | CY157521.1 | *NP* | NCBI |
| A/Turkey/California/189/66(H9N2) | AF156415.1 | *NP* | NCBI |
| A/turkey/Wisconsin/66(H9N2) | DQ067440.1 | *NP* | NCBI |
| A/chicken/Egypt/A18504/2020(H9N2) | ON374923.1 | *NP* | NCBI |
| A/Anhui-Langya/1144/2022(H9N2) | EPI2632297 | *NP* | GISAID |
| A/Cambodia/AHC220057/2022(H9N2) | EPI2436178 | *NP* | GISAID |
| A/Cambodia/21030317/2021(H9N2) | EPI2436170 | *NP* | GISAID |
| A/Bean Goose (Anser fabalis)/South Korea/KNU2021-39/2021(H9N2) | EPI2153549 | *NP* | GISAID |
| A/common teal/Shanghai/NH111143/2021(H9N2) | EPI2058272 | *NP* | GISAID |
| A/layer/Niger/13-23 23VIR3551-13/2023(H9N2) | EPI2550308 | *NP* | GISAID |
| A/broiler/Niger/35-23 23VIR3551-20/2023(H9N2) | EPI2550332 | *NP* | GISAID |
| A/Chicken/Bangladesh/55069/2022(H9N2) | EPI2376701 | *NP* | GISAID |
| A/chicken/Egypt/Menoufia/2021(H9N2) | EPI2175835 | *NP* | GISAID |
| A/chicken/Yunnan/11.22_DQWGH007-O/2018(mixed) | MW099964.1 | *NA* | NCBI |
| A/chicken/Vietnam/HU9-567/2018(H9N2) | LC497163.1 | *NA* | NCBI |
| A/duck/Guangdong/FS91/2022(H9N2) | OQ826096.1 | *NA* | NCBI |
| A/chicken/Shandong/127/2021(H9N2) | MZ703145.1 | *NA* | NCBI |
| A/chicken/Jiangsu/WJ57/2012(H9N2) | KP893706.1 | *NA* | NCBI |
| A/chicken/Jilin/GYH1-1/2012(H9N2) | KF886499.1 | *NA* | NCBI |
| A/Duck/Hong Kong/Y280/97(H9N2) | AF156394.1 | *NA* | NCBI |
| A/chicken/Guangdong/SS/94(H9N2) | DQ874395.1 | *NA* | NCBI |
| A/Chicken/Shanghai/F/98(H9N2) | AY253754.1 | *NA* | NCBI |
| A/Chicken/Beijing/1/94(H9N2) | AF156398.1 | *NA* | NCBI |
| A/environment/Chile/C21617/2017(H9N2) | MH517585.1 | *NA* | NCBI |
| A/northern pintail/California/2789/2011(H9N2) | CY157520.1 | *NA* | NCBI |
| A/American wigeon/Mexico-Sonora/769/2008(H9N2) | KY575198.1 | *NA* | NCBI |
| A/Duck/Hong Kong/Y439/97(H9N2) | AF156395.1 | *NA* | NCBI |
| A/Turkey/California/189/66(H9N2) | AF156401.1 | *NA* | NCBI |
| A/turkey/Wisconsin/66(H9N2) | DQ067439.1 | *NA* | NCBI |
| A/mallard/Alberta/11/1991(H9N2) | CY014591.1 | *NA* | NCBI |
| A/duck/Hokkaido/26/99(H9N2) | AY330339.1 | *NA* | NCBI |
| A/Korea/KBNP-0028/2000(H9N2) | EF620902.1 | *NA* | NCBI |
| A/chicken/Egypt/A18504/2020(H9N2) | ON374924.1 | *NA* | NCBI |
| A/Quail/Hong Kong/G1/97(H9N2) | AF156396.1 | *NA* | NCBI |
| A/chicken/Heilongjiang/35/00(H9N2) | DQ064420.1 | *NA* | NCBI |
| A/Chicken/Hong Kong/G9/97(H9N2) | AF156391.1 | *NA* | NCBI |
| A/Cambodia/AHC220057/2022(H9N2) | EPI2436179 | *NA* | GISAID |
| A/Anhui-Langya/1144/2022(H9N2) | EPI2632298 | *NA* | GISAID |
| A/common teal/Shanghai/NH111143/2021(H9N2) | EPI2058270 | *NA* | GISAID |
| A/Bean Goose (Anser fabalis)/South Korea/KNU2021-39/2021(H9N2) | EPI2153550 | *NA* | GISAID |
| A/chicken/Egypt/Menoufia/2021(H9N2) | EPI2175833 | *NA* | GISAID |
| A/Chicken/Bangladesh/55069/2022(H9N2) | EPI2376702 | *NA* | GISAID |
| A/layer/Niger/13-23 23VIR3551-13/2023(H9N2) | EPI2550314 | *NA* | GISAID |
| A/broiler/Niger/35-23 23VIR3551-20/2023(H9N2) | EPI2550338 | *NA* | GISAID |
| A/Cambodia/21030317/2021(H9N2) | EPI2436171 | *NA* | GISAID |
| A/environment/Yunnan/12.22_DQBHZ001-E/2018(H9N2) | MW101177.1 | *M* | NCBI |
| A/chicken/Shandong/049/2020(H9N2) | MZ703051.1 | *M* | NCBI |
| A/duck/Guangdong/FS91/2022(H9N2) | OQ826097.1 | *M* | NCBI |
| A/chicken/Jiangsu/WJ57/2012(H9N2) | KP893707.1 | *M* | NCBI |
| A/chicken/Guangdong/2.06_SZBJ009-O/2018(H9N2) | MW100830.1 | *M* | NCBI |
| A/chicken/Shandong/127/2021(H9N2) | MZ703146.1 | *M* | NCBI |
| A/chicken/Guangxi/55/2005(H9N2) | EU086248.1 | *M* | NCBI |
| A/chicken/Egypt/A18504/2020(H9N2) | ON374925.1 | *M* | NCBI |
| A/chicken/Jilin/GYH1-1/2012(H9N2) | KF886500.1 | *M* | NCBI |
| A/Chicken/Shanghai/F/98(H9N2) | AY253755.1 | *M* | NCBI |
| A/Chicken/Hong Kong/G9/97(H9N2) | AF156458.1 | *M* | NCBI |
| A/Chicken/Shandong/6/96(H9N2) | AF508700.1 | *M* | NCBI |
| A/Duck/Hong Kong/Y280/97(H9N2) | AF156461.1 | *M* | NCBI |
| A/Chicken/Beijing/1/94(H9N2) | AF156466.1 | *M* | NCBI |
| A/American wigeon/Mexico-Sonora/769/2008(H9N2) | KY575199.1 | *M* | NCBI |
| A/northern pintail/California/2789/2011(H9N2) | CY157519.1 | *M* | NCBI |
| A/environment/New Jersey/UGAI16-0887/2016(H9N2) | CY240898.1 | *M* | NCBI |
| A/turkey/Wisconsin/66(H9N2) | DQ067438.1 | *M* | NCBI |
| A/mallard/Alberta/11/1991(H9N2) | CY005148.1 | *M* | NCBI |
| A/Duck/Hong Kong/Y439/97(H9N2) | AF156462.1 | *M* | NCBI |
| A/Cambodia/21030317/2021(H9N2) | EPI2436172 | *M* | GISAID |
| A/Anhui-Langya/1144/2022(H9N2) | EPI2632299 | *M* | GISAID |
| A/Cambodia/AHC220057/2022(H9N2) | EPI2436180 | *M* | GISAID |
| A/layer/Niger/13-23 23VIR3551-13/2023(H9N2) | EPI2550310 | *M* | GISAID |
| A/broiler/Niger/35-23 23VIR3551-20/2023(H9N2) | EPI2550334 | *M* | GISAID |
| A/Chicken/Bangladesh/55069/2022(H9N2) | EPI2376703 | *M* | GISAID |
| A/chicken/Egypt/Menoufia/2021(H9N2) | EPI2175834 | *M* | GISAID |
| A/chicken/Vietnam/HU9-506/2018(H9N2) | LC497141.1 | *NS* | NCBI |
| A/chicken/Shandong/049/2020(H9N2) | MZ703052.1 | *NS* | NCBI |
| A/chicken/Shandong/3424/2016(H9N2) | MH667576.1 | *NS* | NCBI |
| A/duck/Guangdong/FS91/2022(H9N2) | OQ826098.1 | *NS* | NCBI |
| A/chicken/China/384/2017(H9N2) | MN385392.1 | *NS* | NCBI |
| A/chicken/Guangdong/9.28_SZBJ021-O/2017(H9N2) | MW102262.1 | *NS* | NCBI |
| A/chicken/Shandong/127/2021(H9N2) | MZ703147.1 | *NS* | NCBI |
| A/chicken/Jiangsu/WJ57/2012(H9N2) | KP893708.1 | *NS* | NCBI |
| A/Chicken/Shanghai/F/98(H9N2) | AY253756.1 | *NS* | NCBI |
| A/chicken/Guangxi/55/2005(H9N2) | EU086256.1 | *NS* | NCBI |
| A/chicken/Heilongjiang/35/00(H9N2) | DQ064474.1 | *NS* | NCBI |
| A/chicken/Jilin/GYH1-1/2012(H9N2) | KF886501.1 | *NS* | NCBI |
| A/chicken/Shandong/1/2008(H9N2) | JQ904464.1 | *NS* | NCBI |
| A/Chicken/Hong Kong/G9/97(H9N2) | AF156472.2 | *NS* | NCBI |
| A/Duck/Hong Kong/Y280/97(H9N2) | AF156475.2 | *NS* | NCBI |
| A/Chicken/Beijing/1/94(H9N2) | AF156480.1 | *NS* | NCBI |
| A/quail/Zhejiang/2A3/2013(H9N2) | KU042465.1 | *NS* | NCBI |
| A/quail/Shantou/11195/2005(H9N2) | EF155266.1 | *NS* | NCBI |
| A/Quail/Hong Kong/G1/97 (H9N2) | AF156477.2 | *NS* | NCBI |
| A/chicken/Egypt/Menoufia/2021(H9N2) | EPI2175836 | *NS* | NCBI |
| A/chicken/Egypt/A18504/2020(H9N2) | ON374926.1 | *NS* | NCBI |
| A/Duck/Hong Kong/Y439/97(H9N2) | AF156476.2 | *NS* | NCBI |
| A/Korea/KBNP-0028/2000(H9N2) | EF620904.1 | *NS* | NCBI |
| A/American wigeon/Mexico-Sonora/769/2008(H9N2) | KY575200.1 | *NS* | NCBI |
| A/environment/New Jersey/UGAI16-0887/2016(H9N2) | CY240903.1 | *NS* | NCBI |
| A/mallard/Alberta/11/1991(H9N2) | CY005150.1 | *NS* | NCBI |
| A/turkey/Wisconsin/66(H9N2) | DQ067441.1 | *NS* | NCBI |
| A/Cambodia/AHC220057/2022(H9N2) | EPI2436181 | *NS* | GISAID |
| A/Anhui-Langya/1144/2022(H9N2) | EPI2632296 | *NS* | GISAID |
| A/Cambodia/21030317/2021(H9N2) | EPI2436173 | *NS* | GISAID |
| A/common teal/Shanghai/NH111143/2021(H9N2) | EPI2058273 | *NS* | GISAID |
| A/layer/Niger/13-23 23VIR3551-13/2023(H9N2) | EPI2550309 | *NS* | GISAID |
| A/broiler/Niger/35-23 23VIR3551-20/2023(H9N2) | EPI2550333 | *NS* | GISAID |
| A/Chicken/Bangladesh/55069/2022(H9N2) | EPI2376704 | *NS* | GISAID |

^1^ The sequence information was obtained from the NCBI Nucleotide database or GISAID EpiFluTM database. All submitters of data may be contacted directly via www.gisaid.org or www.ncbi.nlm.nih.gov.

# Supplementary Table S2. Strains exhibiting the highest sequence homology to the isolates identified in this study via NCBI BLASTX.

| **Virus** | **Gene segment** | **Similar virus strains in databases** | **Nucleotide identity (%)** |
| --- | --- | --- | --- |
| CC-3 | *PB2* | A/chicken/Beijing/243/2010(H9N2) | 98.42 |
|  | *PB1* | A/Pigeon/Nanchang/2-0461/2000(H9N2) | 98.14 |
|  | *PA* | A/great bustard/Inner Mongolia/IM-E2/2012(H9N2) | 98.84 |
|  | *HA* | A/chicken/Jilin/GYH1-1/2012(H9N2) | 99.63 |
|  | *NP* | A/chicken/Guangxi/10/99(H9N2) | 99.68 |
|  | *NA* | A/chicken/Jilin/GYH1-1/2012(H9N2) | 99.79 |
|  | *M* | A/chicken/Jilin/GYH1-1/2012(H9N2) | 98.35 |
|  | *NS* | A/chicken/Jilin/GYH1-1/2012(H9N2) | 98.35 |
| CC-6 | *PB2* | A/chicken/Beijing/243/2010(H9N2) | 98.79 |
|  | *PB1* | A/Pigeon/Nanchang/2-0461/2000(H9N2) | 98.19 |
|  | *PA* | A/great bustard/Inner Mongolia/IM-E2/2012(H9N2) | 98.89 |
|  | *HA* | A/chicken/Jilin/GYH1-1/2012(H9N2) | 99.30 |
|  | *NP* | A/chicken/Guangxi/10/99(H9N2) | 99.68 |
|  | *NA* | A/chicken/Jilin/GYH1-1/2012(H9N2) | 99.86 |
|  | *NS* | A/chicken/Jilin/GYH1-1/2012(H9N2) | 99.76 |
| CC-7 | *PB2* | A/chicken/Beijing/243/2010(H9N2) | 98.90 |
|  | *PB1* | A/Pigeon/Nanchang/2-0461/2000(H9N2) | 98.06 |
|  | *PA* | A/great bustard/Inner Mongolia/IM-E2/2012(H9N2) | 99.08 |
|  | *HA* | A/chicken/Jilin/GYH1-1/2012(H9N2) | 99.33 |
|  | *NP* | A/chicken/Guangxi/10/99(H9N2) | 99.68 |
|  | *NA* | A/chicken/Jilin/GYH1-1/2012(H9N2) | 97.00 |
|  | *M* | A/chicken/Jilin/GYH1-1/2012(H9N2) | 95.36 |
|  | *NS* | A/chicken/Shandong/1/2008(H9N2) | 93.53 |
| BHG-8 | *PB2* | A/chicken/Guangdong/1.25_SZBJ010-O/2018(H9N2) | 98.88 |
|  | *PB1* | A/chicken/China/20201202C01/2020(H9N2) | 99.12 |
|  | *PA* | A/duck/Guangdong/8.30_DGCP06-O/2017(H9N2) | 99.32 |
|  | *HA* | A/chicken/Guizhou/03.31_ZYLJJ007-O/2018(H9N2) | 98.46 |
|  | *NP* | A/chicken/Shandong/10.23_TAWL020-O/2018(H9N2) | 99.24 |
|  | *NA* | A/chicken/Yunnan/11.22_DQWGH007-O/2018(mixed) | 98.86 |
|  | *M* | A/chicken/China/HN22/2022(H9N2) | 99.60 |
|  | *NS* | A/chicken/Guangdong/9.28_SZBJ021-O/2017(H9N2) | 98.81 |
| CC-11 | *PB2* | A/chicken/Beijing/243/2010(H9N2) | 99.18 |
|  | *PB1* | A/Pigeon/Nanchang/2-0461/2000(H9N2) | 98.19 |
|  | *PA* | A/great bustard/Inner Mongolia/IM-E2/2012(H9N2) | 99.16 |
|  | *HA* | A/chicken/Jilin/GYH1-1/2012(H9N2) | 97.00 |
|  | *NP* | A/great bustard/Inner Mongolia/IM-E2/2012(H9N2) | 95.19 |
|  | *NA* | A/chicken/Jilin/GYH1-1/2012(H9N2) | 99.79 |
|  | *M* | A/chicken/Jilin/GYH1-1/2012(H9N2) | 98.04 |
|  | *NS* | A/chicken/Jilin/GYH1-1/2012(H9N2) | 98.94 |
| BHG-21 | *PB2* | A/chicken/Beijing/243/2010(H9N2) | 98.86 |
|  | *PB1* | A/Pigeon/Nanchang/2-0461/2000(H9N2) | 97.86 |
|  | *PA* | A/great bustard/Inner Mongolia/IM-E2/2012(H9N2) | 96.83 |
|  | *NP* | A/chicken/Guangxi/10/99(H9N2) | 99.22 |
|  | *NA* | A/chicken/Jilin/GYH1-1/2012(H9N2) | 99.86 |
|  | *NS* | A/chicken/Jilin/GYH1-1/2012(H9N2) | 99.76 |
| CK-54 | *PB2* | A/chicken/Guangdong/1.25_SZBJ010-O/2018(H9N2) | 98.91 |
|  | *PB1* | A/chicken/Xinjiang/09.19_WLMQXL026-O/2018(mixed) | 99.12 |
|  | *PA* | A/duck/Guangdong/8.30_DGCP06-O/2017(H9N2) | 99.32 |
|  | *HA* | A/duck/China/D4/2018(H9N2) | 98.70 |
|  | *NP* | A/chicken/Shandong/10.23_TAWL025-O/2018(H9N2) | 99.27 |
|  | *NA* | A/chicken/Yunnan/11.22_DQWGH007-O/2018(mixed) | 98.86 |
|  | *M* | A/chicken/Guangdong/2.06_SZBJ009-O/2018(H9N2) | 99.50 |
|  | *NS* | A/chicken/Guangdong/9.28_SZBJ021-O/2017(H9N2) | 98.58 |
| CK-59 | *PB2* | A/chicken/Guangdong/1.25_SZBJ010-O/2018(H9N2) | 98.91 |
|  | *PB1* | A/chicken/Xinjiang/09.19_WLMQXL026-O/2018(mixed) | 98.37 |
|  | *PA* | A/chicken/China/93/2017(H9N2) | 98.19 |
|  | *HA* | A/chicken/Guizhou/03.31_ZYLJJ007-O/2018(H9N2) | 98.10 |
|  | *NP* | A/chicken/Guangxi/8.25_NNXH008-O/2018(mixed) | 98.33 |
|  | *NA* | A/chicken/Vietnam/HU9-567/2018(H9N2) | 97.97 |
|  | *M* | A/chicken/Guangdong/2.06_SZBJ009-O/2018(H9N2) | 99.50 |
|  | *NS* | A/chicken/China/384/2017(H9N2) | 97.69 |
|  | *NS* | A/chicken/Vietnam/HU9-506/2018(H9N2) | 99.31 |
| CK-68 | *PB2* | A/chicken/Shandong/097/2020(H9N2) | 98.95 |
|  | *PB1* | A/chicken/China/1102/2019(H9N2) | 99.31 |
|  | *PA* | A/chicken/Anhui/LH99/2017(H9N2) | 98.37 |
|  | *HA* | A/duck/China/D4/2018(H9N2) | 98.70 |
|  | *NP* | A/chicken/Shandong/10.23_TAWL020-O/2018(H9N2) | 99.20 |
|  | *NA* | A/chicken/Yunnan/11.22_DQWGH007-O/2018(mixed) | 95.90 |
|  | *M* | A/chicken/China/HN22/2022(H9N2) | 99.60 |
|  | *NS* | A/chicken/China/384/2017(H9N2) | 95.84 |
| CK-74 | *PB2* | A/chicken/Vietnam/HU9-506/2018(H9N2) | 98.62 |
|  | *PB1* | A/chicken/Yunnan/12.22_DQXBL001-O/2018(H9N2) | 97.63 |
|  | *PA* | A/chicken/Vietnam/HU9-447/2018(H9N2) | 99.05 |
|  | *HA* | A/chicken/Anhui/AJ35/2017(H9N2) | 96.97 |
|  | *NP* | A/chicken/Guangxi/8.25_NNXH008-O/2018(mixed) | 98.92 |
|  | *NA* | A/chicken/Yunnan/11.22_DQWGH007-O/2018(mixed) | 97.86 |
|  | *M* | A/duck/Hubei/S0895/2015(H9N2) | 97.05 |
|  | *NS* | A/chicken/Shandong/3424/2016(H9N2) | 98.84 |
| CK-75 | *PB2* | A/chicken/Vietnam/HU9-506/2018(H9N2) | 99.09 |
|  | *PB1* | A/chicken/China/FY110/2022(H9N2) | 98.68 |
|  | *PA* | A/chicken/Hunan/12.27_YYGK13G3-OC/2017(H9N2) | 98.84 |
|  | *HA* | A/gallus/China/Hefei/2017(H9N2) | 98.07 |
|  | *NP* | A/chicken/Yunnan/11.22_DQWGH007-O/2018(mixed) | 98.06 |
|  | *NA* | A/chicken/Yunnan/11.22_DQWGH007-O/2018(mixed) | 98.22 |
|  | *M* | A/environment/Yunnan/12.22_DQBHZ001-E/2018(H9N2) | 98.40 |





**Supplementary Figure S1.** Phylogenetic analysis of the gene segments of polymerase basic protein 2 (*PB2*), polymerase basic protein 1 (*PB1*), polymerase acidic (*P*A), nucleoprotein (*NP*), neuraminidase (NA), matrix proteins (*M*), and non-structural proteins (*NS*). The phylogenetic tree is generated with IQ-TREE software employing the maximum likelihood (ML) method. Node support is evaluated through 1,000 bootstrap replicates, and support values exceeding 70% are indicated at the corresponding nodes. The eleven H9N2 strains sequenced in this study are denoted by red triangles, while reference sequences for each clade are emphasized in bold. Branch-specific nomenclature is detailed to the right of the tree.
